# Comprehensive characterization of micropapillary colorectal adenocarcinoma

**DOI:** 10.1002/path.6392

**Published:** 2025-02-07

**Authors:** Ville K Äijälä, Jouni Härkönen, Tuomo Mantere, Hanna Elomaa, Päivi Sirniö, Vesa‐Matti Pohjanen, Onni Sirkiä, Henna Karjalainen, Meeri Kastinen, Vilja V Tapiainen, Sara A Väyrynen, Petri Pölönen, Maarit Ahtiainen, Olli Helminen, Erkki‐Ville Wirta, Jukka Rintala, Sanna Meriläinen, Juha Saarnio, Tero Rautio, Katri Pylkäs, Toni T Seppälä, Jan Böhm, Jukka‐Pekka Mecklin, Anne Tuomisto, Markus J Mäkinen, Juha P Väyrynen

**Affiliations:** ^1^ Translational Medicine Research Unit Medical Research Center Oulu, Oulu University Hospital, and University of Oulu Oulu Finland; ^2^ Department of Pathology Hospital Nova of Central Finland, Well Being Services County of Central Finland Jyväskylä Finland; ^3^ Faculty of Health Sciences A.I. Virtanen Institute for Molecular Sciences, University of Eastern Finland Kuopio Finland; ^4^ Laboratory of Cancer Genetics and Tumor Biology, Translational Medicine Research Unit Medical Research Center Oulu and Biocenter Oulu, University of Oulu Oulu Finland; ^5^ Department of Biological and Environmental Science University of Jyväskylä Jyväskylä Finland; ^6^ Department of Education and Research Well Being Services County of Central Finland Jyväskylä Finland; ^7^ Department of Environmental and Biological Sciences University of Eastern Finland Kuopio Finland; ^8^ Department of Internal Medicine Oulu University Hospital Oulu Finland; ^9^ Department of Pathology St. Jude Children's Research Hospital Memphis TN USA; ^10^ Department of Surgery Oulu University Hospital Oulu Finland; ^11^ Department of Gastroenterology and Alimentary Tract Surgery Tampere University Hospital Tampere Finland; ^12^ Faculty of Medicine and Health Technology Tampere University and Tays Cancer Centre, Tampere University Hospital Tampere Finland; ^13^ Northern Finland Laboratory Centre Nordlab Oulu Finland; ^14^ Department of Gastrointestinal Surgery Helsinki University Central Hospital, University of Helsinki Helsinki Finland; ^15^ Applied Tumor Genomics, Research Program Unit University of Helsinki Helsinki Finland; ^16^ Faculty of Sport and Health Sciences University of Jyväskylä Jyväskylä Finland

**Keywords:** micropapillary, colorectal cancer, prognosis, immunology, multiplex immunohistochemistry, bioinformatics, optical genome mapping, epithelial‐mesenchymal transition

## Abstract

Micropapillary colorectal adenocarcinoma is a morphologic subtype of colorectal cancer (CRC) with insufficiently characterized prognostic significance and biological features. We analyzed the histopathological, immunological, and prognostic features of micropapillary adenocarcinoma in two independent CRC cohorts (*N* = 1,876). We found that micropapillary adenocarcinomas accounted for 4.9% and 6.4% of CRCs in the two cohorts. A micropapillary growth pattern was associated with advanced stage and lymphovascular invasion (*p* < 0.001), but also with shorter overall survival independent of these factors and other prognostic parameters (Cohort 1: hazard ratio [HR] 1.76, 95% confidence interval [CI] 1.08–2.87; Cohort 2: HR 1.47, 95% CI 1.08–2.00). Multiplex immunohistochemistry and machine learning‐assisted image analysis showed that the micropapillary growth pattern was associated with decreased CD3^+^ T‐cell and CD14^+^HLA‐DR^+^ monocytic cell densities. Molecular features of micropapillary adenocarcinoma were studied using bioinformatic analyses in The Cancer Genome Atlas (TCGA) cohort (*N* = 629) and validated with optical genome mapping and immunohistochemistry. These analyses revealed that micropapillary adenocarcinomas frequently present with chromosome region 8q24 copy number gain, *TP53* mutation, and overexpression of *UPK2, MUC16*, and epithelial‐mesenchymal transition involved genes, such as *L1CAM*. These results indicate that micropapillary colorectal adenocarcinoma is an aggressive morphologic subtype of CRC characterized by shorter overall survival, decreased antitumorigenic immune response, and unique molecular features. Our findings support the classification of micropapillary adenocarcinoma as a distinct, high‐risk subtype of CRC, which should be systematically evaluated in patient care. © 2025 The Author(s). *The Journal of Pathology* published by John Wiley & Sons Ltd on behalf of The Pathological Society of Great Britain and Ireland.

## Introduction

Colorectal cancer (CRC) is the third most common cancer in the world and the second leading cause of cancer death [1]. Over 90% of CRCs are adenocarcinomas [2], which can be further classified into morphologic subtypes with varying histopathological, molecular, and prognostic features [3], including signet‐ring cell, mucinous, serrated, medullary, and micropapillary carcinomas [4]. Molecular alterations contribute to the heterogenous nature of CRC [5]. Microsatellite instability (MSI), in particular, appears to be frequently present in medullary and mucinous CRCs [2, 6, 7] and *BRAF* mutation is highly present in serrated and medullary carcinomas [8]. However, the molecular background of many growth patterns is currently unknown.

**Figure 1:**
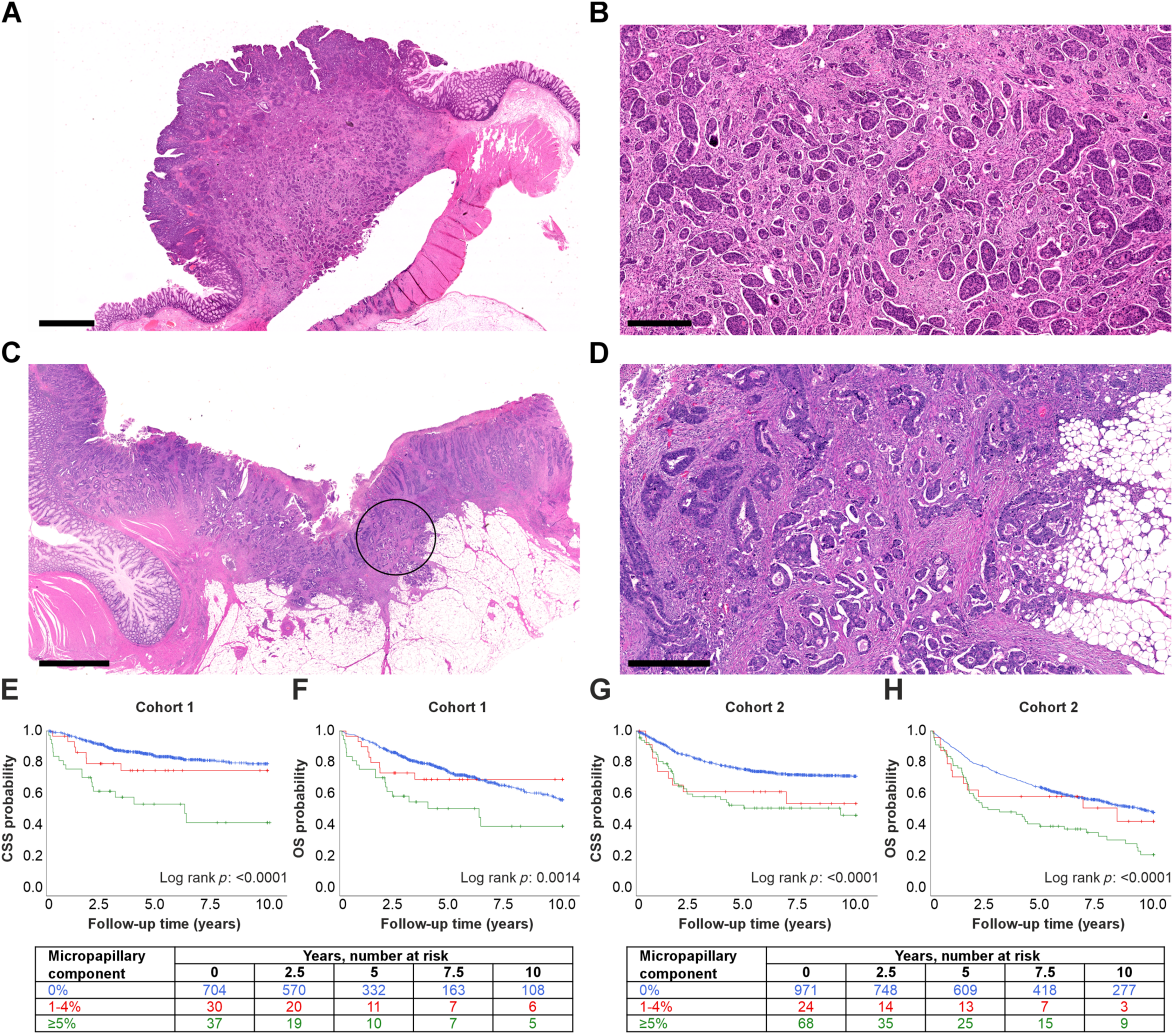
Histological features and Kaplan–Meier survival analyses of micropapillary colorectal adenocarcinoma. (A) A hematoxylin & eosin (H&E)‐stained section showing the micropapillary growth pattern in most of the tumor. (B) Close‐up magnification of tumor A. The growth pattern is composed of reverse polarity tumor cell clusters that are frequently surrounded by retraction artifacts. (C) An H&E‐stained section of a tumor with a smaller micropapillary component indicated by a circle. (D) Close‐up magnification of tumor C. Micropapillary structures are surrounded by tumor cells arranged in glandular structures without micropapillary features. (E and F) The association of a micropapillary component with cancer‐specific survival (E) and overall survival (F) in Cohort 1. (G and H) The association of the micropapillary component with cancer‐specific survival (G) and overall survival (H) in Cohort 2. Scale bar, 2.5 mm (A and C) and 500 μm (B and D). Abbreviations: CSS, cancer‐specific survival; OS, overall survival.

Micropapillary adenocarcinoma is characterized by a reverse polarity of small, often eosinophilic tumor cell clusters, frequently surrounded by lacunae (Figure 1A–D) [9]. It is a well‐recognized subtype of various cancers, including breast [10], bladder [11], and lung cancer [12], but micropapillary CRC has not been extensively investigated [9]. A few studies have linked micropapillary CRC with adverse prognosis, although there are some conflicting results regarding its independent prognostic effect [9, 13, 14, 15, 16]. In various organs, micropapillary adenocarcinomas differ from conventional adenocarcinomas in terms of somatic mutations, as well as mRNA, miRNA, and protein expression [17]. While micropapillary CRC has been reported to show altered expression of villin [18] and RHOA [19] and higher mutation frequency of *TP53* [20], among others, its molecular profile remains largely unknown. Further research is needed to elucidate both the molecular features and the prognostic significance of micropapillary CRC, potentially enabling more precise patient care.

The purpose of this study was to comprehensively characterize micropapillary CRC in terms of its histopathological, immunological, and molecular features, as well as prognostic significance. To accomplish these aims, we first defined the characteristics of micropapillary CRCs in two large CRC cohorts (*N* = 1,876) with extensive clinicopathologic annotation. We then screened for the molecular features characteristic of micropapillary CRC in publicly available data (*N* = 629) and validated several key features in our own cohort.

## Materials and methods

### Ethical considerations

The study was performed in accordance with the Helsinki Declaration. For Cohort 1, the study was conducted under approval from the Regional Medical Research Ethics Committee of the Wellbeing services county of North Ostrobothnia (25/2002, 42/2005, 122/2009, 37/2020), Biobank Borealis (BB‐2017_1012), and Fimea (FIMEA/2022/001941). For Cohort 2, the study was conducted under approval from the Regional Medical Research Ethics Committee of the Wellbeing services county of Central Finland (13U/2011, 1/2016, 8/2020, 2/2023), Central Finland Biobank (BB23‐0172), and Fimea (FIMEA/2023/001573). In Cohort 1, all participants gave written informed consent for the study. For Cohort 2, the need to obtain informed consent from the study patients was waived (FIMEA/2023/001573, 4/2023).

### Study population

Three independent cohorts were analyzed (supplementary material, Figure S1).

Cohort 1 consisted of stage I–IV colorectal cancer patients (*N* = 1,011) surgically treated in Oulu University Hospital between 2006 and 2020. Cohort 2 consisted of colorectal cancer patients (*N* = 1,343) surgically treated in the Central Hospital of Central Finland between 2000 and 2015. The Cancer Genome Atlas (TCGA) cohort (*N* = 629) was used for the analysis of the molecular features of micropapillary CRCs.

Patients who had received preoperative radiotherapy or chemoradiotherapy were excluded from the analyses (Cohort 1: *N* = 235; Cohort 2: *N* = 243), considering potential effects of neoadjuvant treatments on tumor histology. Furthermore, patients who died within 30 days of surgery were excluded from survival analyses (Cohort 1: *N* = 5; Cohort 2: *N* = 37). In total, data from 776 or 771 patients in Cohort 1 and 1,100 or 1,063 patients in Cohort 2 could be utilized.

### Histopathological analyses

Tumor morphologic subtypes were visually estimated from hematoxylin & eosin‐stained whole‐slide digital images. While the WHO tumor classification [2] sets the criterion for micropapillary colorectal adenocarcinoma as a micropapillary component comprising ≥5% of the tumor surface area, our study evaluated the micropapillary component percentage as a continuous variable and categorized it to groups of 0%, 1%–4%, and ≥5% to enable investigation of a lesser micropapillary component. For Cohort 2, all cases were reviewed by VKÄ and JPV. The reproducibility kappa score for the evaluations of ≥5% versus <5% micropapillary percentage was 0.71. Any discrepancies were resolved through consensus discussion. For Cohort 1 and the TCGA cohort, cases with a micropapillary component were screened by VKÄ and confirmed by JPV. In the TCGA breast cancer cohort, a micropapillary growth pattern was independently evaluated by JPV and VMP, and discordant cases were resolved through consensus discussion. pTNM stage was determined by the UICC (Union for International Cancer Control) criteria [21]. Tumor budding was evaluated using the guidelines recommended by the International Tumor Budding Consensus Conference [22], and poorly differentiated clusters were evaluated in a similar manner by counting the numbers of poorly differentiated tumor cell clusters (five tumor cells or more) in hotspots (0.785 mm^2^) and categorizing these into low (<5), intermediate (5–9), and high (≥10). Lymphovascular invasion was also evaluated on hematoxylin & eosin‐stained tumor sections and was defined as tumor cells within vascular spaces. Lymphocytic reaction patterns (tumor‐infiltrating lymphocytes, intratumoral periglandular reaction, peritumoral reaction, and Crohn's‐like lymphoid reaction) were evaluated according to the criteria suggested by Ogino *et al* [23] and categorized into absent, low, intermediate, and high. All histopathological parameters were collected blinded to the survival outcome.

### Immunohistochemistry

Tissue microarrays were used in immunohistochemistry. They were designed to include four 1‐mm‐diameter cores per tumor (two from the tumor center and two from the invasive margin) [24, 25]. Regions with a micropapillary growth pattern were not specifically targeted. HER2 immunohistochemistry and *in situ* hybridization were performed using a Roche (Indianapolis, IN, USA) Ventana Benchmark Ultra. For HER2 immunohistochemistry, 4B5 antibody (Roche 790‐4493, prediluted) was used with Ventana CC1 (Roche 950‐224, 8 min, 95 °C) and Ventana UltraView Universal DAB Detection Kit (Roche 760‐500). For *HER2 in situ* hybridization, Ventana HER2 Dual ISH DNA Probe Cocktail (Roche 800‐6043, denaturation time 8 min at 80 °C, hybridization time 60 min at 44 °C) was used with Ventana CC2 (950‐123, 8 min, 82 °C), Ventana Protease 3 (760‐2020, 20 min 36 °C). Other immunohistochemistry was performed using Leica (Wetzlar, Germany) Bond RX or Leica Bond 3 automated stainers and the BOND Polymer Refine Detection kit (Leica DS9800) and BOND Epitope Retrieval Solution 2 (Leica AR9640, 30 min, 100 °C). Antibodies used included those to detect TP53 (Epredia, Kalamazoo, MI, USA; clone DO‐7, 1:200), UPK2 (Biocare, Pacheco, CA, USA; clone BC21, 1:50), MUC16 (CA125) (BioLegend, San Diego, CA, USA; clone 618F, 1:100), L1CAM (BioLegend, clone 14.10, 1:50), and MYC (Cell Marque, Rocklin, CA, USA; clone EP121, 1:30).

HER2 immunohistochemistry and *in situ* hybridization were scored according to the criteria established for gastroesophageal adenocarcinoma (supplementary material, Figure S2) [26]. Although these guidelines indicate that HER2 *in situ* hybridization is required only for tumors with an HER2 immunohistochemistry score of 2+ [26], *in situ* hybridization was performed for all tumors in this study. Tumors were considered positive for *HER2* amplification if they had an *HER2*:*CEP17* signal ratio ≥2 or average *HER2* copy number ≥6 signals per cell. TP53 expression in tumor cells was categorized into mutation‐pattern (diffuse overexpression or absent), and wildtype‐pattern (heterogeneous). Expression levels of UPK2 (cytoplasmic), L1CAM (membranous), MUC16 (membranous), and MYC (nuclear) were evaluated as the percentage of positive tumor cells. Considering the overall low expression rate of UPK2, L1CAM, and MUC16 in CRC, dichotomous variables (negative, 0% versus positive >0%) were used in downstream analyses. MYC expression was classified into low (≤50%) versus high (>50%) using the median as the cut‐point. Mismatch repair (MMR) status and *BRAF* V600E mutation status were evaluated as described previously [24].

### Immune cell analyses

**Figure 2:**
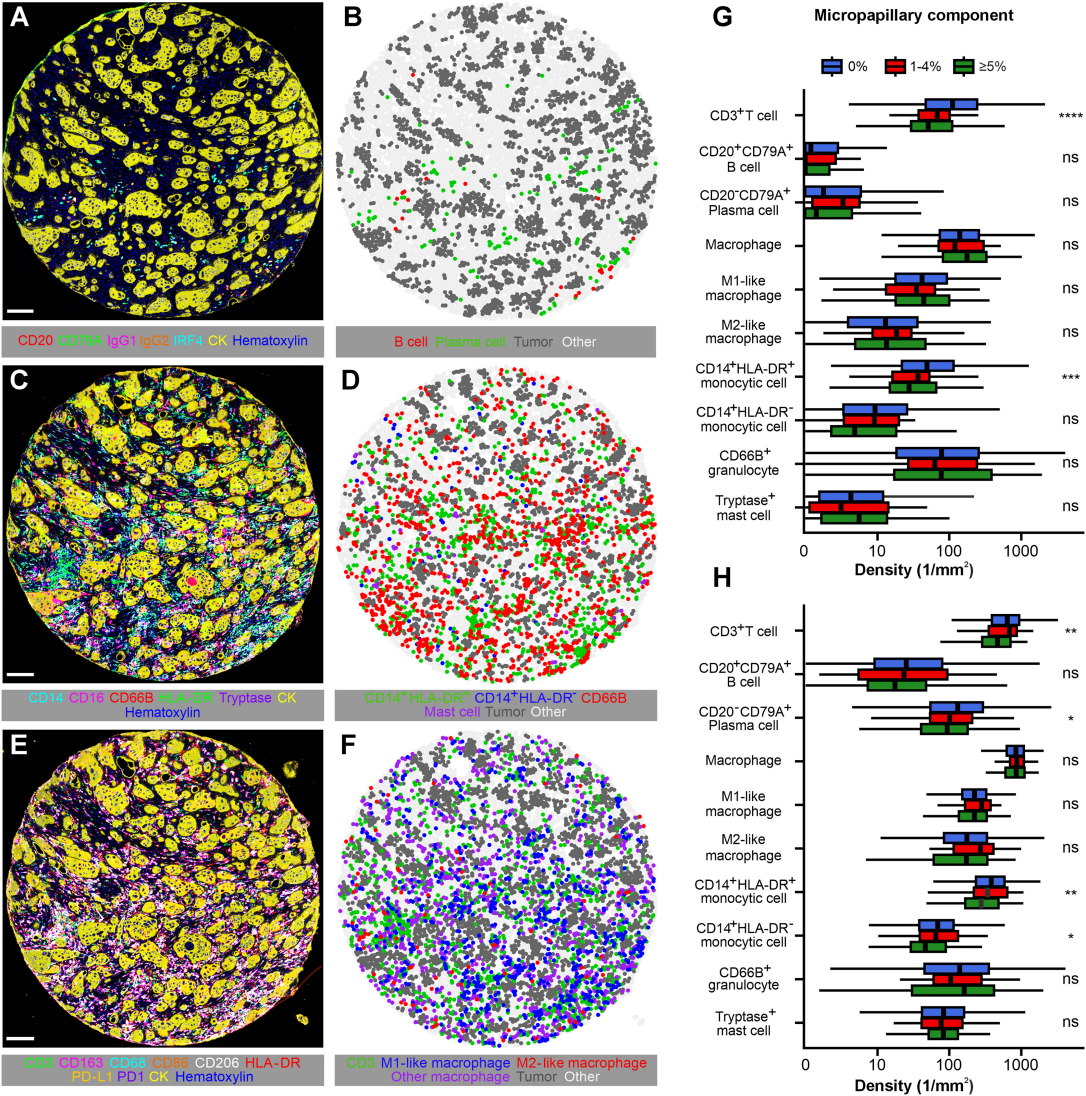
Multiplex‐immunohistochemistry panel, image analysis, and immune cell density analysis. (A–E) Example multiplex immunohistochemistry images of a micropapillary colorectal adenocarcinoma (A, C and E) and their corresponding cell maps (B, D and F) based on machine learning assisted image analyses. (G and H) Boxplots of distributions of immune cell densities in the tumor intraepithelial region (G) and tumor stroma (H) according to the micropapillary growth pattern. The analyses are based on Cohort 2: *N* = 1,065 for CD3^+^ T‐cells, macrophages, M1‐like macrophages, and M2‐like macrophages; *N* = 1,045 for CD14^+^HLA‐DR^+^ mature monocytic cells, CD14^+^HLA‐DR^−^ immature monocytic cells, CD66B^+^ granulocytes, and tryptase^+^ mast cells; *N* = 1,070 for CD20^+^CD79A^+^ B cells and CD20^−^CD79A^+^ plasma cells. *p value <0.05, **p value <0.01, ***p value <0.001, ****p value <0.0001. Scale bars, 100 μm.

Immune cell densities of tumor samples were evaluated with tissue microarrays using multiplex immunohistochemistry combined with digital image analysis (Figure 2A–F). The assays were based on a cyclic method with 3‐Amino‐9‐ethylcarbazole as the chromogen, described and validated previously in detail (supplementary material, Figure S3) [27, 28]. Cell densities were separately evaluated in the tumor intraepithelial and stromal regions. The cell types analyzed in this study included CD3^+^ T‐cells, CD20^+^CD79A^+^ B cells, CD20^−^CD79A^+^ plasma cells, M1‐like and M2‐like macrophages, CD14^+^HLA‐DR^+^ mature monocytic cells, CD14^+^HLA‐DR^−^ immature monocytic cells, CD66B^+^ granulocytes, and tryptase^+^ mast cells. Macrophage phenotypes were defined by a polarization index based on four polarization markers (M1: CD86, HLA‐DR; M2: CD163, CD206) [27].

### Bioinformatic analyses

Preprocessed TCGA‐data and genome‐wide segment files were downloaded from https://gdc.cancer.gov/node/905/ (EBPlusPlus gene expression and mutation) and https://gdac.broadinstitute.org/runs/stddata__2016_01_28/data/ (segmented scna minus germline against hg18 for copy‐number, as well as RSEM‐counts for consensus molecular subtyping).

CRC segment files were split to represent micropapillary and other samples, and GISTIC (Genomic Identification of Significant Targets In Cancer) v.2.0 run was conducted with standard settings using the hg18 reference matrix. Breast cancer copy number variation was assessed with the same parameters for the whole dataset. Copy number data were visualized with maftools v.2.12.0. For differential gene expression, samples having more than 5,000 NA values were filtered out. To address negative values in the preprocessed TCGA data, a pseudocount of 1 was added to the linear gene expression values, after which the data were reverted to log2 space. Differential gene expression analysis for micropapillary vs. other cases was computed with limma 3.58.14 [29] according to the user manual (*Linear Models for Microarray and RNA‐Seq Data User's Guide page 71, 22/04/23*). Gene set enrichment analysis (GSEA) was conducted with the fGSEA‐package for the log fold‐change‐ranked gene list, using GSEA‐MSigDB hallmark and positional gene set collections. CMScaller [30] was conducted for the TCGA RSEM‐counts as recommended by the function description (https://rdrr.io/github/peterawe/CMScaller/man/CMScaller.html). Heatmaps were visualized with ComplexHeatmap v.2.13.2.

Tumor mutational burden was computed from the maf file as the number of mutations/Mb for each sample by assuming 30 Mb coverage (size of the human exome). Silent variants were discarded for the subsequent analyses. The maximum value from MLH1‐methylation probe *β*‐values was used as the methylation metric for each sample.

For the breast cancer cohort, a gene expression score for chr8q24 locus was defined as mean log2 mRNA expression of the leading‐edge genes at the respective locus in the CRC DE‐analysis (supplementary material, Table S1).

### Optical genome mapping

Optical genome mapping experiments were conducted in accordance with the manufacturer's instructions using the Saphyr instrument and DLE‐1 chemistry (Bionano Genomics, San Diego, CA, USA). Ultrahigh‐molecular‐weight (UHMW) genomic DNA was extracted using the SP Tissue and Tumor DNA Isolation Kit (#80038). For this, approximately 10 mg of fresh‐frozen tumor tissue was cut and placed in the homogenization buffer, and the homogenization process was performed using TissueRuptor (Qiagen, Chatsworth, CA, USA). The extracted UHMW genomic DNA was labeled with the Direct Label and Stain technique following the manufacturer's protocol (Bionano Prep DLS Labeling Kit; Bionano Genomics) with kit version G1 or G2. Subsequently, the fluorescent‐labeled UHMW genomic DNA samples were loaded onto Saphyr chips (G2.3 or G3.3) and run on the Saphyr instrument. The amount of data to be collected was set to 1,800 Gbp, and GRCh38/hg38 was utilized as the reference genome.

Optical genome mapping data analysis was conducted using the rare variant pipeline (RVP) included in Bionano Solve software (Bionano Genomics, v.3.8) and visualized in Bionano Access software (Bionano Genomics, v.1.8.1), using the default confidence scores and size‐cutoffs for structural variants, aneuploidy, and copy number variation calling. All structural variants present in the OGM control database (303 individuals) provided by Bionano Genomics were filtered out.

### Statistical analyses

Statistical analyses were performed using IBM SPSS Statistics for Windows (IBM v.29.0, Armonk, NY, USA) or R statistical programming (v.4.3.3, Vienna, Austria). Findings with two‐tailed *p* < 0.05 were considered statistically significant.

Associations of micropapillary CRC categories with patient and tumor characteristics were studied using crosstabulation, and the 𝜒^2^ test was used in evaluating the statistical significance. The Kaplan–Meier method and Cox proportional hazards regression models were used to evaluate the association of micropapillary CRC categories with cancer‐specific survival (CSS) and overall survival (OS), defined as time from surgery to cancer death or end of follow‐up and time from surgery to death or end of follow‐up, respectively. The follow‐up was limited to 10 years, considering that most CRC deaths occur within 10 years. The median follow‐up time for censored cases was 5.6 years (interquartile range [IQR] 3.7–9.3) in Cohort 1 and 10.0 years (IQR 7.3–10.0) in Cohort 2. Multivariable Cox proportional hazards regression models included the following covariates: age (<65, 65–75, >75), sex (male, female), year of operation (Cohort 1: 2006–2010, 2011–2015, 2016–2020; Cohort 2: 2000–2005, 2006–2010, 2011–2015), tumor location (proximal colon, distal colon, rectum), disease stage (I–II, III, IV), lymphovascular invasion (no, yes), MMR status (MMR‐proficient, MMR‐deficient), *BRAF* status (wildtype, mutant), and *HER2* amplification (no, yes). Cases with missing data for *BRAF* status (seven cases in Cohort 1; two cases in Cohort 2) and *HER2* amplification (16 cases in Cohort 1; 24 cases in Cohort 2) were incorporated into the majority category (*BRAF* wildtype; *HER2* no amplification) to limit the degrees of freedom.

In bioinformatic analyses, Fisher's tests and Mann–Whitney *U* tests were utilized to evaluate statistical significance for categorical‐categorical and categorical‐continuous variable comparisons, respectively. For COAD & READ cohort gene expression, the false‐discovery rate (FDR) values (BH‐corrected) from the differential expression analysis were used as indicators of statistical significance. For the *a priori* defined mRNA features in the breast cancer‐validation analysis, multiple‐comparison correction for statistical testing was performed with the Bonferroni method.

## Results

### Clinicopathologic features

The characteristics of 1,876 CRC cases according to the presence of micropapillary component are summarized in Table 1. There were 68 (8.8%) and 97 (8.8%) tumors with a micropapillary component in Cohorts 1 and 2, respectively. In these tumors, the micropapillary component comprised, on average, 10% of the tumor (SD: 13%, range: 1%–60%) in Cohort 1% and 15% (SD: 19%, range: 1%–85%) in Cohort 2 (supplementary material, Figure S4). Micropapillary adenocarcinoma (micropapillary component ≥5%) accounted for 4.9% and 6.4% of CRCs in the two cohorts. The micropapillary growth pattern was associated with advanced disease stage, nodal and distant metastasis, lymphovascular invasion, tumor budding, and poorly differentiated clusters in both cohorts (*p* ≤ 0.001 for all). The micropapillary growth pattern was associated with *HER2* amplification in Cohort 2 (*p* = 0.0039) but not in Cohort 1 (*p* = 0.33).

**Table 1:** Patient and tumor characteristics and their associations with the micropapillary growth pattern in cohorts 1 and 2

	Cohort 1					Cohort 2				
	Total *N*	Micropapillary growth pattern			*p* value	Total *N*	Micropapillary growth pattern			*p* value
Characteristic		0%	1%–4%	≥5%			0%	1%–4%	≥5%	
All cases	776 (100%)	708 (91%)	30 (3.9%)	38 (4.9%)		1,100 (100%)	1,003 (91%)	27 (2.5%)	70 (6.4%)	
Sex					0.81					0.43
Female	364 (47%)	330 (91%)	16 (4.4%)	18 (4.9%)		543 (49%)	497 (92%)	10 (1.8%)	36 (6.6%)	
Male	412 (53%)	378 (92%)	14 (3.4%)	20 (4.9%)		557 (51%)	506 (91%)	17 (3.1%)	34 (6.1%)	
Age (years)					0.26					0.38
<65	233 (30%)	213 (91%)	11 (4.7%)	9 (3.9%)		290 (26%)	256 (88%)	10 (3.4%)	24 (8.3%)	
65–75	285 (37%)	254 (89%)	11 (3.9%)	20 (7.0%)		381 (35%)	351 (92%)	8 (2.1%)	22 (5.8%)	
>75	258 (33%)	241 (93%)	8 (3.1%)	9 (3.5%)		429 (39%)	396 (92%)	9 (2.1%)	24 (5.6%)	
Year of operation					**0.028**					0.55
2000–2005	‐	‐	‐	‐		342 (31%)	312 (91%)	6 (1.8%)	24 (7.0%)	
2006–2010	155 (20%)	132 (85%)	10 (6.5%)	13 (8.4%)		353 (32%)	317 (90%)	12 (3.4%)	24 (6.8%)	
2011–2015	218 (28%)	206 (94%)	4 (1.8%)	8 (3.7%)		405 (37%)	374 (92%)	9 (2.2%)	22 (5.4%)	
2016–2020	403 (52%)	370 (92%)	16 (4.0%)	17 (4.2%)		‐	‐	‐	‐	
Tumor location					0.32					0.80
Proximal colon	323 (42%)	291 (90%)	11 (3.4%)	21 (6.5%)		536 (49%)	486 (91%)	12 (2.2%)	38 (7.1%)	
Distal colon	205 (26%)	191 (93%)	9 (4.4%)	5 (2.4%)		404 (37%)	369 (91%)	12 (3.0%)	23 (5.7%)	
Rectum	248 (32%)	226 (91%)	10 (4.0%)	12 (4.8%)		160 (15%)	148 (93%)	3 (1.9%)	9 (5.6%)	
UICC disease stage					**<0.0001**					**<0.0001**
I	189 (24%)	183 (97%)	3 (1.6%)	3 (1.6%)		184 (17%)	179 (97%)	0 (0.0%)	5 (2.7%)	
II	251 (32%)	240 (96%)	7 (2.8%)	4 (1.6%)		408 (37%)	390 (96%)	5 (1.2%)	13 (3.2%)	
III	251 (32%)	217 (86%)	17 (6.8%)	17 (6.8%)		355 (32%)	307 (86%)	15 (4.2%)	33 (9.3%)	
IV	85 (11%)	68 (80%)	3 (3.5%)	14 (16%)		153 (14%)	127 (83%)	7 (4.6%)	19 (12%)	
T					**0.013**					**0.0086**
T1‐T2	229 (30%)	219 (96%)	6 (2.6%)	4 (1.7%)		225 (20%)	217 (96%)	2 (0.9%)	6 (2.7%)	
T3‐T4	547 (70%)	489 (89%)	24 (4.4%)	34 (6.2%)		875 (80%)	786 (90%)	25 (2.9%)	64 (7.3%)	
N					**<0.0001**					**<0.0001**
N0	455 (59%)	437 (96%)	10 (2.2%)	8 (1.8%)		626 (57%)	601 (96%)	7 (1.1%)	18 (2.9%)	
N1‐N2	321 (41%)	271 (84%)	20 (6.2%)	30 (9.3%)		474 (43%)	402 (85%)	20 (4.2%)	52 (11%)	
M					**<0.0001**					**0.0011**
M0	691 (89%)	640 (93%)	27 (3.9%)	24 (3.5%)		947 (86%)	876 (93%)	20 (2.1%)	51 (5.4%)	
M1	85 (11%)	68 (80%)	3 (3.5%)	14 (16%)		153 (14%)	127 (83%)	7 (4.6%)	19 (12%)	
Lymphovascular invasion					**<0.0001**					**<0.0001**
No	429 (55%)	410 (96%)	10 (2.3%)	9 (2.1%)		858 (78%)	809 (94%)	17 (2.0%)	32 (3.7%)	
Yes	347 (45%)	298 (86%)	20 (5.8%)	29 (8.4%)		242 (22%)	194 (80%)	10 (4.1%)	38 (16%)	
Tumor budding					**<0.0001**					**<0.0001**
Grade 1 (0–4)	541 (70%)	526 (97%)	8 (1.5%)	7 (1.3%)		827 (75%)	803 (97%)	11 (1.3%)	13 (1.6%)	
Grade 2 (5–9)	129 (17%)	111 (86%)	9 (7.0%)	9 (7.0%)		156 (14%)	129 (83%)	7 (4.5%)	20 (13%)	
Grade 3 (≥10)	106 (14%)	71 (67%)	13 (12%)	22 (21%)		117 (11%)	71 (61%)	9 (7.7%)	37 (32%)	
Poorly differentiated clusters					**<0.0001**					**<0.0001**
0–4	624 (80%)	612 (98%)	7 (1.1%)	5 (0.8%)		894 (81%)	877 (98%)	10 (1.1%)	7 (0.8%)	
5–9	73 (9.4%)	60 (82%)	8 (11%)	5 (6.8%)		114 (10%)	92 (81%)	9 (7.9%)	13 (11%)	
≥10	79 (10%)	36 (46%)	15 (19%)	28 (35%)		92 (8.4%)	34 (37%)	8 (8.7%)	50 (54%)	
MMR status					0.90					0.091
Proficient	652 (84%)	594 (91%)	25 (3.8%)	33 (5.1%)		931 (85%)	842 (90%)	26 (2.8%)	63 (6.8%)	
Deficient	124 (16%)	114 (92%)	5 (4.0%)	5 (4.0%)		169 (15%)	161 (95%)	1 (0.6%)	7 (10%)	
*BRAF* status*					0.90					0.22
Wild‐type	662 (86%)	605 (91%)	25 (3.8%)	32 (4.8%)		916 (83%)	840 (92%)	23 (2.5%)	53 (5.8%)	
Mutant	107 (14%)	96 (90%)	5 (4.7%)	6 (5.6%)		182 (17%)	161 (88%)	4 (2.2%)	17 (9.3%)	
*HER2* amplification^†^					0.33					**0.0039**
No	723 (95%)	657 (91%)	28 (3.9%)	38 (5.3%)		1,044 (97%)	954 (91%)	22 (2.1%)	68 (6.5%)	
Yes	37 (4.9%)	35 (95%)	2 (5.4%)	0 (0.0%)		32 (3.0%)	28 (88%)	4 (13%)	0 (0.0%)	

*Note*: *p* values in bold are statistically significant.

Abbreviations: MMR, mismatch repair; UICC, Union for International Cancer Control.

* Data missing for seven cases in Cohort 1 and 2 cases in Cohort 2.

^†^ Data missing for 16 cases in Cohort 1 and 24 cases in Cohort 2.

### Survival

In 10‐year survival analyses, there were 244 deaths (135 cancer deaths) in Cohort 1 and 531 deaths (296 cancer deaths) in Cohort 2. In univariable analysis, the micropapillary growth pattern was associated with shorter CSS and OS in both cohorts (Figure 1E–H, Table 2). In multivariable analysis, the micropapillary growth pattern remained independently associated with shorter CSS in Cohort 1 (*p*_trend_ = 0.0002) and shorter OS in both cohorts (Cohort 1: *p*_trend_ = 0.018; Cohort 2: *p*_trend_ = 0.019). The multivariable hazard ratio (HR) for micropapillary component ≥5% (vs. 0%) was 1.76 (95% confidence interval [CI] 1.08–2.87) in Cohort 1 and 1.47 (95% CI 1.08–2.00) in Cohort 2 (Table 2 and supplementary material, Table S2).

**Table 2:** Cox proportional hazards regression models for colorectal cancer‐specific survival and overall survival according to micropapillary growth pattern in Cohort 1 and 2

Variable	No. of cases	Cancer‐specific survival			Overall survival		
		No. of events	Univariable HR (95% CI)	Multivariable HR (95% CI)	No. of events	Univariable HR (95% CI)	Multivariable HR (95% CI)
**Cohort 1**							
Micropapillary growth pattern							
0%	704	110	1 (referent)	1 (referent)	216	1 (referent)	1 (referent)
1%–4%	30	7	1.69 (0.79–3.63)	1.88 (0.85–4.13)	9	1.093 (0.56–2.13)	1.36 (0.69–2.68)
≥5%	37	18	4.26 (2.59–7.02)	2.57 (1.52–4.32)	19	2.33 (1.45–3.72)	1.76 (1.08–2.87)
*p*_trend_			**<0.0001**	**0.0002**		**0.001**	**0.018**
**Cohort 2**							
Micropapillary growth pattern							
0%	971	254	1 (referent)	1 (referent)	470	1 (referent)	1 (referent)
1%–4%	24	10	1.92 (1.02–3.61)	1.11 (0.58–2.12)	12	1.29 (0.73–2.28)	0.96 (0.53–1.72)
≥5%	68	32	2.32 (1.60–3.35)	1.24 (0.84–1.82)	49	2.04 (1.52–2.75)	1.47 (1.08–2.00)
*p*_trend_			**<0.0001**	0.26		**<0.0001**	**0.019**

*Note*: Multivariable Cox proportional hazards regression models were adjusted for sex, age (<65, 65–75, >75), year of operation (Cohort 1: 2006–2010, 2011–2015, 2016–2020; Cohort 2: 2000–2005, 2011–2015, 2016–2020), tumor location (proximal colon, distal colon, rectum), disease stage (I–II, III, IV), lymphovascular invasion (no, yes), mismatch repair (MMR) status (proficient, deficient), *BRAF* status (wildtype, mutant), *HER2* amplification (no, yes). *p* values in bold are statistically significant.

Abbreviations: CI, confidence interval; HR, hazard ratio.

### Immune cells

Considering that the micropapillary growth pattern has been associated with immunosuppression and immune evasion in lung [31] and breast cancer [32], we next assessed whether immune cell infiltration patterns might differ according to the presence of the micropapillary component in CRC (Figure 2G,H). The micropapillary growth pattern was associated with weaker lymphocytic reactions, as evaluated using hematoxylin & eosin‐stained sections (*p* < 0.01 for all four patterns) (supplementary material, Table S3). In more detailed analyses utilizing multiplex immunohistochemistry, the micropapillary growth pattern was associated with decreased CD3^+^ T‐cell and CD14^+^HLA‐DR^+^ monocytic cell densities both in the tumor intraepithelial and stromal regions. Additionally, there was an association between the micropapillary growth pattern and decreased CD20^−^CD79A^+^ plasma cell and CD14^+^HLA‐DR^−^ monocytic cell densities within the tumor stroma.

### Molecular characteristics

**Figure 3:**
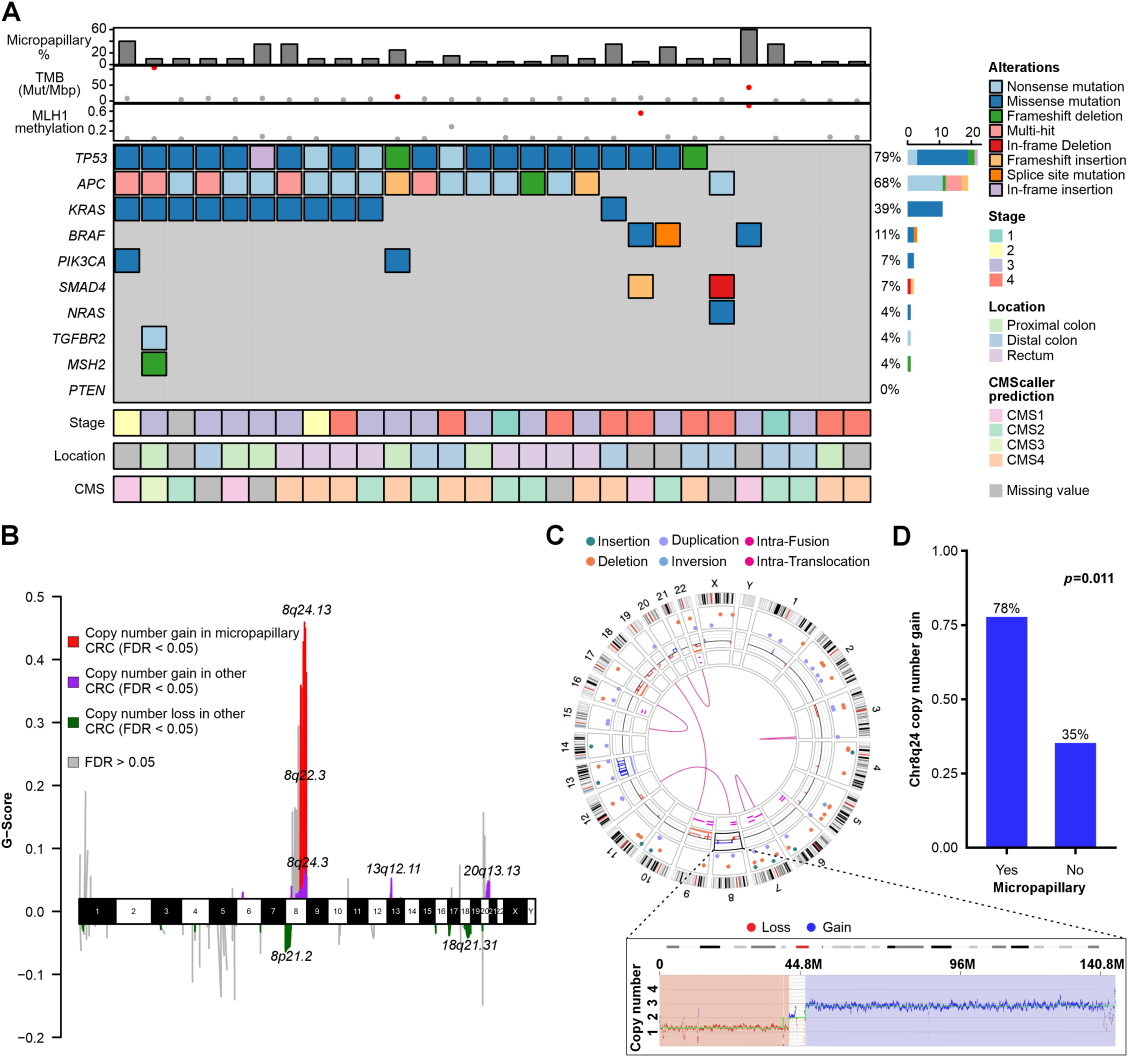
Somatic mutations and copy number alterations in micropapillary colorectal adenocarcinoma. (A) Heatmap showing mutation frequency of common colorectal cancer associated mutations in micropapillary colorectal adenocarcinomas of the TCGA cohort (*N* = 28), along with basic clinicopathologic features of the tumors. (B) GISTIC analysis of somatic copy number variation in micropapillary (vs. other) colorectal cancers in the TCGA cohort. The G‐score denotes the amplitude of an aberration along with its frequency across multiple samples. (C) Optical genome mapping Circos plot of a micropapillary colorectal cancer, showing copy number gain of Chr8q with concomitant loss of Chr8p. (D) Bar chart showing frequency of Chr8q24 copy number gain in micropapillary (*N* = 18) and nonmicropapillary (*N* = 17) colorectal cancers using optical genome mapping. CMS, consensus molecular subtype; CRC, colorectal cancer; FDR, false discovery rate; TMB, tumor mutational burden.

In the TCGA Cohort, 28 (4.5%) of tumors were classified as micropapillary adenocarcinomas. The clinical and molecular features of these cases are summarized in Figure 3A. *TP53* mutation was more prevalent in micropapillary CRCs (79%) compared to other cases (60%, *p* = 0.006), while *PIK3CA* was less frequent (7% in micropapillary CRCs versus 25% in other cases, *p* = 0.039). A higher frequency of *TP53* alteration in micropapillary CRC was also validated with immunohistochemistry in Cohort 1, where a mutation‐type staining pattern was observed in 29/38 (76%) micropapillary adenocarcinomas (versus 18/29 [62%] CRCs with micropapillary component, and 389/693 (56%) nonmicropapillary CRCs, *p* = 0.043) (supplementary material, Figure S5).

GISTIC analysis of somatic copy number variation revealed a frequent gain of chromosome 8q in micropapillary adenocarcinomas, most prominent in regions 8q24 and 8q22 (both FDR < 0.05) (Figure 3B). The phenomenon was investigated in more detail using optical genome mapping of 35 cases (18 micropapillary, 17 nonmicropapillary) in Cohort 1 (Figure 3C). This analysis confirmed frequent copy number gain of Chr8q24 in micropapillary CRCs (14/18, 78% of micropapillary cases versus 6/17, 35% in nonmicropapillary, *p* = 0.011) (Figure 3D). Of these cases, whole Chr8q arm (6/14, 43% micropapillary versus 5/6, 83% nonmicropapillary), and whole Chr8 (3/14, 21% micropapillary versus 4/6, 67% nonmicropapillary) copy number gains were more prevalent in nonmicropapillary CRCs. When tumors with copy number gain of whole Chr8 were excluded, 11/15 (73%) micropapillary and 2/13 (15%) nonmicropapillary CRCs showed Chr8q copy number gain. Of the micropapillary CRCs with Chr8q copy number gain, 71% (10/14) showed concomitant Chr8p loss. Chr8q24 copy number gain ranged between 2 and 4 in 11/14 cases and >4 for 3/14 cases. Notably, one tumor showed amplification of *MYC* with copy number above 20. Considering that oncogene *MYC* is located at Chr8q24, we evaluated whether MYC is overexpressed in micropapillary carcinomas using immunohistochemistry in Cohort 1 (supplementary material, Figure S6). However, our results showed no statistically significant associations between MYC expression and a micropapillary growth pattern (*p* = 0.17). *MYC* mRNA was not overexpressed in micropapillary CRCs in the TCGA cohort, either (FDR = 0.98).

Considering the morphologic overlap between micropapillary CRC and micropapillary carcinomas of other organs, we investigated whether the same molecular features that we observed in micropapillary CRC might characterize micropapillary carcinomas of the breast. We calculated the Chr8q24 score (based on the mean expression levels of leading‐edge DE‐genes in the CRC‐analysis from the same region) for breast cancers in the TCGA breast cancer Cohort (*N* = 1,098) and evaluated the top and bottom 4% of cases for the presence of a micropapillary growth pattern (supplementary material, Figure S7). Within the top 4% of breast cancers, nine (20%) cases were morphologically classified as invasive micropapillary carcinomas of the breast, while no micropapillary carcinomas were found in the bottom 4% (*p* = 0.002). The top 4% of breast cancers had an overrepresentation of *TP53* mutations (*p* < 0.0001) and an underrepresentation *PIK3CA* mutations (*p* = 0.046), also corresponding to the molecular features that we observed in micropapillary CRC.

**Figure 4:**
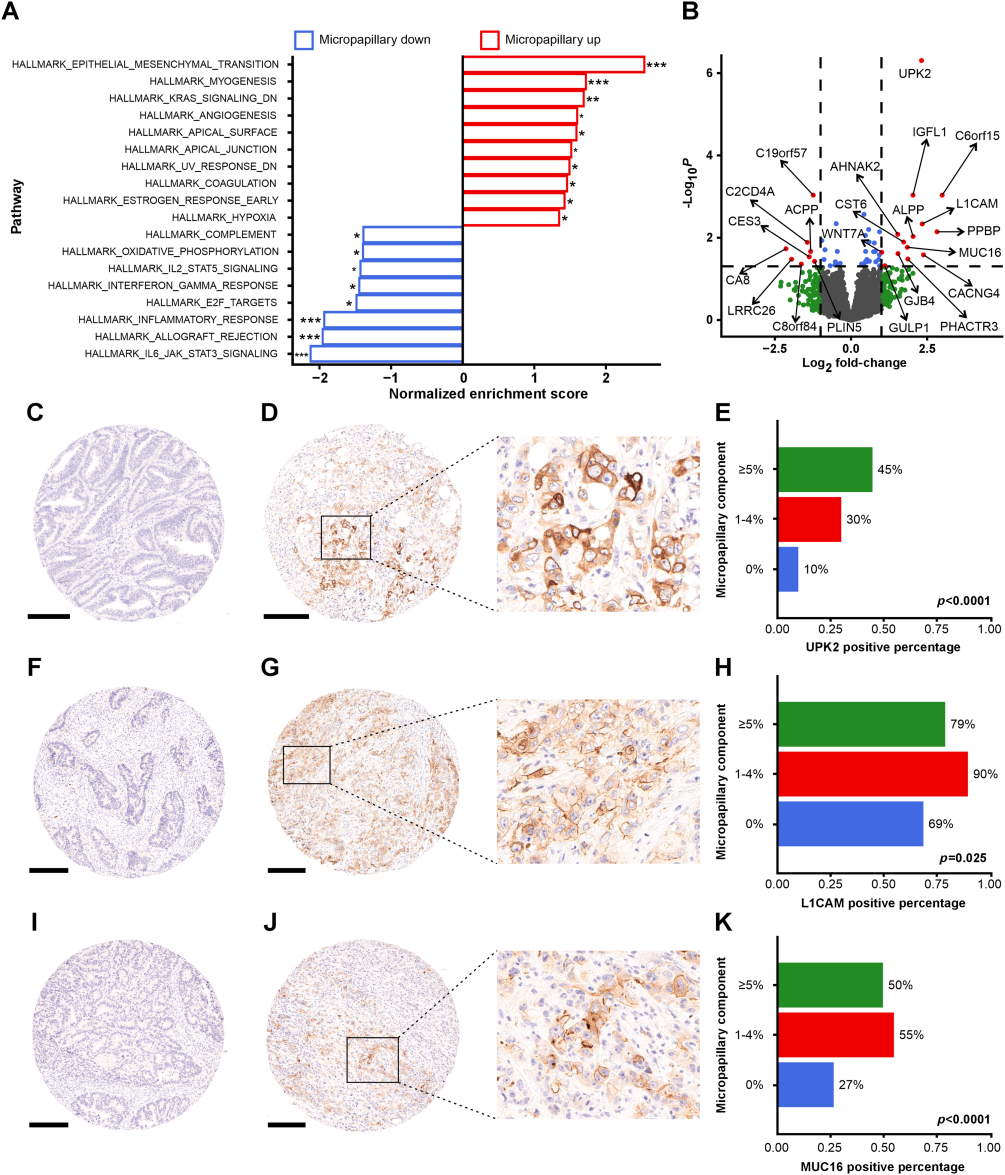
Gene expression patterns in micropapillary colorectal adenocarcinoma. (A) Gene set enrichment analysis of micropapillary (vs. other) colorectal adenocarcinoma. (B) Volcano plot of the differentially expressed genes in micropapillary colorectal adenocarcinoma. (C and D) Example tissue microarray cores of UPK2 staining with a negative sample (C) and positive sample showing cytoplasmic staining (D). (F and G) Example tissue microarray cores of L1CAM staining with a negative sample (F) and positive sample showing membranous staining (G). (I and J) Example tissue microarray cores of MUC16 (CA125) staining with a negative sample (I) and positive sample showing membranous staining (J). (E, H and K) Bar charts depicting the increased expression of UPK2 (E), L1CAM (H), and MUC16 (K) in colorectal cancers with a micropapillary component. (A) and (B) are based on the TCGA cohort (*N* = 629). C–K are based on Cohort 1 [*N* = 761 (UPK2), *N* = 760 (L1CAM and MUC16)]. *p value <0.05, **p value <0.01, ***p value <0.001. Scale bar, 250 μm.

Finally, we analyzed mRNA expression in TCGA micropapillary CRCs. Gene set enrichment analysis of micropapillary (≥5% versus other) CRCs showed positive enrichment scores for epithelial‐mesenchymal transition (EMT) and myogenesis, among others (Figure 4A). Negative enrichment scores were observed primarily for hallmarks related to immune reactions such as IL6/JAK/STAT3 signaling. The single genes that were differentially expressed are shown in Figure 4B. Of these genes, we chose three, *UPK2*, *L1CAM*, and *MUC16* (*CA125*) for further investigation using immunohistochemistry in Cohort 1, based on the availability of well‐validated antibodies against these targets. These proteins were mainly expressed on tumor epithelium, and CRCs with a micropapillary growth pattern showed higher expression of all three proteins (Figure 4C–K), with the most notable difference in UPK2 expression (*p* < 0.0001).

## Discussion

The purpose of this study was to characterize the histopathological, immunological, molecular, and prognostic features of micropapillary CRC, utilizing two cohorts with more than 1,800 cases, as well as the TCGA cohort. Micropapillary adenocarcinoma was associated with shorter CSS and OS in both cohorts. The micropapillary growth pattern was negatively correlated with densities of antitumorigenic immune cell subtypes. At the molecular level, micropapillary CRC was characterized by chromosome 8q copy number gain (most prominent at 8q24), *TP53* mutation, lower *PIK3CA* mutation frequency, and overexpression of genes associated with EMT. These findings reveal novel biological features of micropapillary CRC and support the classification of micropapillary adenocarcinoma as a unique, high‐risk CRC subtype.

The micropapillary growth pattern was present in around 9% of CRC cases and micropapillary CRC, defined by micropapillary component ≥5%, represented 4.9% and 6.4% of all cases in Cohorts 1 and 2, respectively. Other studies have generally reported incidences ranging between 5% and 20% [2]. A micropapillary growth pattern was associated with several parameters indicating poor prognosis such as advanced disease stage and lymphovascular invasion, but it also represented an independent prognostic factor for OS in both cohorts. This finding supports the systematic evaluation of the micropapillary growth pattern in patient care. In survival analysis, the cases with micropapillary component of 1%–4% harbored intermediate prognosis, suggesting that there might be some value in including these cases as a separate category when evaluating the micropapillary growth pattern in CRC. Previous studies have had conflicting results regarding the prognostic significance of micropapillary CRC. Xu *et al* [16] found micropapillary CRC to be an independent unfavorable prognostic factor in TNM stages I and II while Lee *et al* [14] and Pyo *et al* [33] determined it to be associated with a significantly shorter OS. On the other hand, some studies have found no effect on survival or the effect limited only to univariable models [9, 15]. Some of the discrepancies between studies could be explained by differences in methodology, as well as the rarity of micropapillary CRC, making it difficult to obtain large enough sample sizes.

Micropapillary CRC was associated with weaker lymphocytic reactions, as well as decreased T‐cell, plasma cell, and monocytic cell (particularly mature monocytic cell) infiltrates at the tumor microenvironment. Few studies have been conducted on the immunological profile of micropapillary CRC. Deshpande *et al* [34] studied immune markers with immunohistochemistry and found micropapillary CRC to be associated with lower CD8^+^ T‐cell counts and β2‐microglobulin and PD‐L1 expression. Using more general parameters such as tumor‐infiltrating lymphocytes, Crohn‐like lymphoid reaction, peritumoral inflammation, and tumor invasive front inflammation, no statistically significant associations were found in previous single studies [9, 16, 20, 35]. Our main analyses were based on multiplex immunohistochemistry, which enables the detection of more detailed cellular phenotypes than conventional single‐plex immunohistochemistry. Despite MMR deficiency being one of the key factors influencing the tumor microenvironment in CRC, as well as molecular pathological features of tumors, we found no significant association between micropapillary CRC and MMR status, suggesting that other factors influence the association of micropapillary CRC with weaker immune reactions. We found many immune cell signaling pathways, such as IL6/JAK/STAT3 signaling, to be negatively enriched in micropapillary CRCs, potentially contributing to the finding.

EMT signaling pathway genes were enriched in micropapillary CRCs. This could account for the association of micropapillary CRC with many parameters linked to cancer invasion. EMT describes the phenomenon in which epithelial cells gain mesenchymal cell‐like properties to enable embryogenetic and malignant processes [36]. EMT is induced by the Wnt pathway and TGF‐β pathway, among others, and it is known to promote increased motility, invasive capacity, reversed cell polarity, and stem cell‐like properties in neoplastic epithelium [36]. Evidence of EMT in micropapillary CRC has been previously reported as the subtype appears to show, for example, abnormal E‐cadherin expression [9, 35, 37] and inversed *MUC1* pattern [37]. Similarly, Lee *et al* [14] showed increased expression of stem cell markers such as *SOX2* in micropapillary CRC. Our results highlighted the EMT marker *L1CAM* as one of the genes highly expressed in micropapillary CRC. Interestingly, *UPK2* (uroplakin 2) was also frequently expressed in micropapillary colorectal adenocarcinoma. UPK2 is a component of urothelial plaques, specialized membrane domains in urothelial superficial (umbrella) cells, and is used as a relatively specific marker for urothelial carcinomas [38, 39]. Considering the primary role of UPK2 as the permeability barrier of the urothelium, we hypothesize that UPK2 could potentially facilitate the formation of micropapillary structures, and MUC16, that we also identified as a marker for micropapillary CRC, could further contribute to the protective epithelial barrier function of micropapillary structures by providing a hydrophilic environment.

One of the most distinct molecular features associated with micropapillary CRCs was Chr8q copy number gain (most prominent at Chr8q24). Chromosome region 8q24 amplification is one of the most common alterations in cancer [40] and variants on this region contribute to the risk of CRC [41]. *MYC* is a proto‐oncogene located in Chr8q24, which may account for the high prevalence of Chr8q24 copy number gain in cancer overall [40, 41]. However, our results show no significant increase of *MYC* mRNA nor protein expression in micropapillary CRC, suggesting that this copy number gain in micropapillary CRCs may more significantly affect some other factors on the 8q24 locus. To investigate the potential role of Chr8q24 copy number gain in micropapillary carcinomas of different organs, we calculated Chr8q24 scores for TCGA breast cancers. We found that micropapillary carcinomas of the breast were overrepresented in cases with high Chr8q24 scores, suggesting that this chromosome region might more generally contribute to the micropapillary features in carcinomas. Amplification of region 8q24 in invasive micropapillary carcinoma of the breast has been previously reported by one study [42] but no similar findings have been reported in micropapillary colorectal adenocarcinoma. *TP53* mutation and lack of *PIK3CA* mutation were also associated with both micropapillary CRCs and higher Chr8q24 scores in breast cancer, further highlighting the similarity of the molecular features of micropapillary carcinomas of these two organs.

Our study has some limitations. The assessment of the micropapillary growth pattern may be subjective. However, each case with a micropapillary component was reviewed by two investigators, and Cohen's kappa between two observers was substantial, supporting the reproducibility of the evaluation. Tissue microarrays, which were used for immune cell density analyses, did not specifically target the micropapillary components of tumors. Therefore, the immune cell findings that we observed were related to the average immune microenvironment of the tumors. However, tissue microarrays enabled us to consistently analyze a large number of samples using three multiplex immunohistochemistry panels. Although our cohorts contained extensive clinicopathologic annotation, data on perineural invasion were not available, and we could not evaluate its potential association with the micropapillary growth pattern. Moreover, data on *KRAS/NRAS* mutation status, which represent important predictive factors for anti‐EGFR treatment, were not available. However, the micropapillary growth pattern was not associated with *KRAS/NRAS* status in the TCGA cohort. We excluded patients who had received neoadjuvant treatment, considering its potential influence on the histological features of CRC. Therefore, it needs to be confirmed whether the results are applicable to neoadjuvant‐treated CRCs. Similarly, we did not examine the effect of the micropapillary growth pattern on patients’ adjuvant treatment response, and further studies should investigate whether patients with micropapillary CRC would benefit from specific treatments. Finally, the sequencing analyses were based on only the TCGA cohort and were limited to bulk DNA and RNA sequencing. Advanced techniques such as spatial transcriptomics and single‐cell RNA sequencing could provide deeper insights into the pathogenetic mechanisms associated with micropapillary colorectal cancer and reveal additional disease biomarkers.

Our study has several strengths. Ours was a large study based on two CRC cohorts with more than 1,800 cases. Most previous studies have been based on 100–1,000 patients. We used multiplex immunohistochemistry to enable more detailed analyses of immune cells in tumors than is possible with traditional immunohistochemistry. Likewise, the molecular data available from TCGA cohort enabled the most detailed molecular characterization of micropapillary CRC done thus far, and we validated several key findings in our own cohort, including detailed characterization of chromosome region 8q alterations using optical genomic mapping, which is a high‐resolution method for evaluating structural variations.

In conclusion, micropapillary CRC is associated with distinct molecular, clinicopathologic, and immunological features, and it represents an independent factor of shorter OS, supporting its categorization as a high‐risk subtype of CRC. These findings could eventually influence the introduction of a more aggressive treatment protocol for patients with micropapillary CRC.

## Author contributions statement

VKÄ, JH, MJM and JPV conceptualized the study. VKÄ, J‐PM, MJM and JPV acquired funding for the project. MJM and JPV supervised the study. VKÄ, JH, HE, PS, OS, HK, MK, VVT, PP, MA, OH, E‐VW, JR, TTS, JB, AT, MJM and JPV curated the data. VKÄ, JH, TM, HE, SAV, KP and JPV contributed to the methodology. VKÄ, JH and JPV performed formal analysis. VKÄ, JH, TM and JPV provided visualization. VKÄ, JH, TM and JPV drafted the manuscript. All authors participated in the investigation, contributed to manuscript editing, and approved the final version of the manuscript.

## Supporting information


**Figure S1.** Flowcharts of the patients and cohorts analyzed in the study
**Figure S2**. *HER2* immunohistochemistry and *in situ* hybridization in colorectal cancer
**Figure S3**. Multiplex immunohistochemistry protocol
**Figure S4**. Distributions of micropapillary growth pattern percentages
**Figure S5**. TP53 alterations in micropapillary colorectal adenocarcinoma
**Figure S6**. MYC expression in micropapillary colorectal adenocarcinoma
**Figure S7**. Chromosome region 8q24 gene expression score analysis of TCGA BRCA cohort cases
**Table S1**. Genes used for the Chr8q24 locus gene expression score in the breast cancer cohort analysis
**Table S2**. Multivariable Cox proportional hazards regression models for cancer‐specific survival and overall survival in Cohort 1 and 2
**Table S3**. Associations of lymphocytic reaction patterns with the micropapillary growth pattern in Cohort 2 (*N* = 1,100)

## Data Availability

TCGA data were downloaded from public repositories (https://gdc.cancer.gov/node/905/ and https://gdac.broadinstitute.org/runs/stddata__2016_01_28/data/). Other data generated and/or analyzed during this study are not publicly available. The sharing of data will require approval from relevant Ethics Committees and/or biobanks. Further information including the procedures to obtain and access data of Finnish Biobanks are described at https://finbb.fi/en/fingenious-service.
