# Supplementary material for: Comprehensive characterization of micropapillary colorectal adenocarcinoma
Source: J Pathol. 2025 Feb 7;265(4):408–21. doi: 10.1002/path.6392 (PMC11880967; doi:10.1002/path.6392)
Supplement: Supplementary file 1 — Figure S1. Flowcharts of the patients and cohorts analyzed in the study Figure S2. HER2 immunohistochemistry and in situ hybridization in colorectal cancer Figure S3. Multiplex immunohistochemistry protocol Figure S4. Distributions of micropapillary growth pattern percentages Figure S5. TP53 alterations in micropapillary colorectal adenocarcinoma Figure S6. MYC expression in micropapillary colorectal adenocarcinoma Figure S7. Chromosome region 8q24 gene expression score analysis of TCGA BRCA cohort cases Table S1. Genes used for the Chr8q24 locus gene expression score in the breast cancer cohort analysis Table S2. Multivariable Cox proportional hazards regression models for cancer‐specific survival and overall survival in Cohort 1 and 2 Table S3. Associations of lymphocytic reaction patterns with the micropapillary growth pattern in Cohort 2 (N = 1,100) [file PATH-265-408-s001.docx]

**Comprehensive characterization of micropapillary colorectal adenocarcinoma**

VK Äijälä *et al. J Pathol* <https://doi.org/10.1002/path.6392>

**Supplementary Figures S1–S7**

**Supplementary Tables S1–S3**


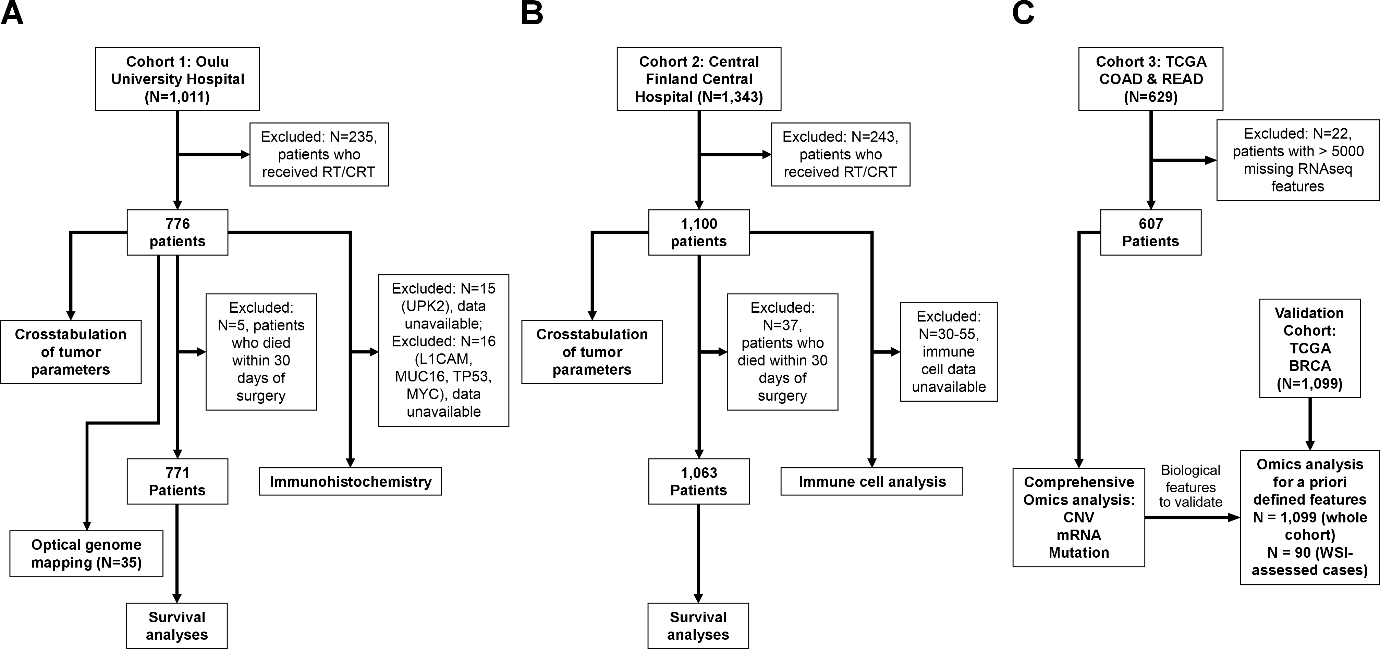


**Figure S1.** Flowcharts of the patients and cohorts analyzed in the study. (A) Flowchart of Cohort 1, which was used for clinicopathologic, survival, and immunohistochemistry analyses. (B) Flowchart of Cohort 2, which was used for validation and immune cell analyses. (C) TCGA cohorts that were used for bioinformatic analyses (COAD & READ) and validation of biological features (BRCA). Abbreviations: RT, radiotherapy; CRT, chemoradiotherapy; TCGA, The Cancer Genome Atlas; CNV, copy number variation

**
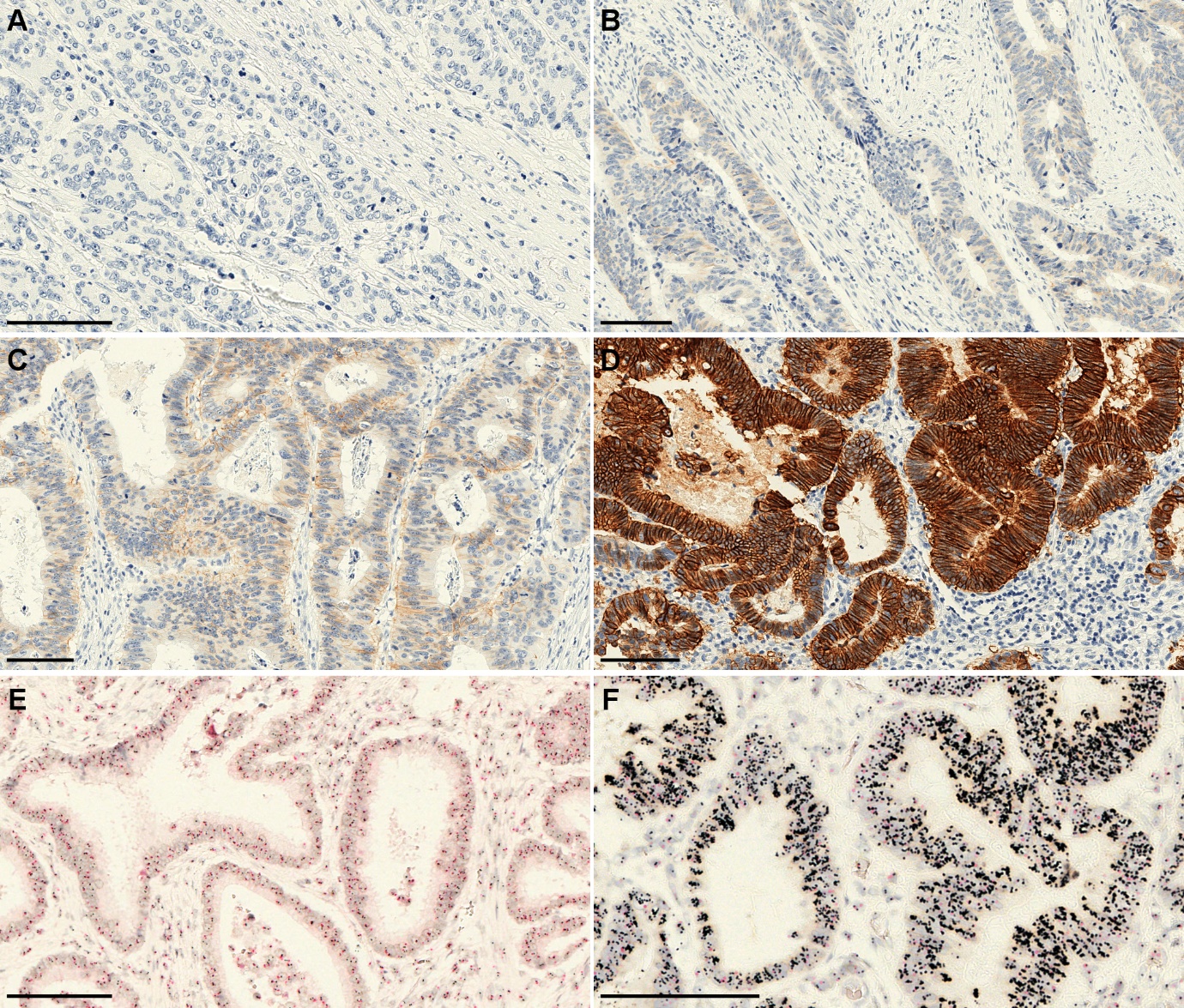
**

**Figure S2.** *HER2* immunohistochemistry and *in situ* hybridization in colorectal cancer. (A–D) Representative cases of colorectal cancer *HER2* immunohistochemistry scored as (A) negative 0, (B) negative 1+, (C) equivocal 2+, or (D) positive 3+ staining. (E) Example of a nonamplified tumor with *HER2*:*CEP17* signal ratio of 1. (F) Example of an amplified tumor with *HER2*:*CEP17* signal ratio >2. Black signals denote *HER2* and red signals *CEP17*. Scale bar, 100 µm.


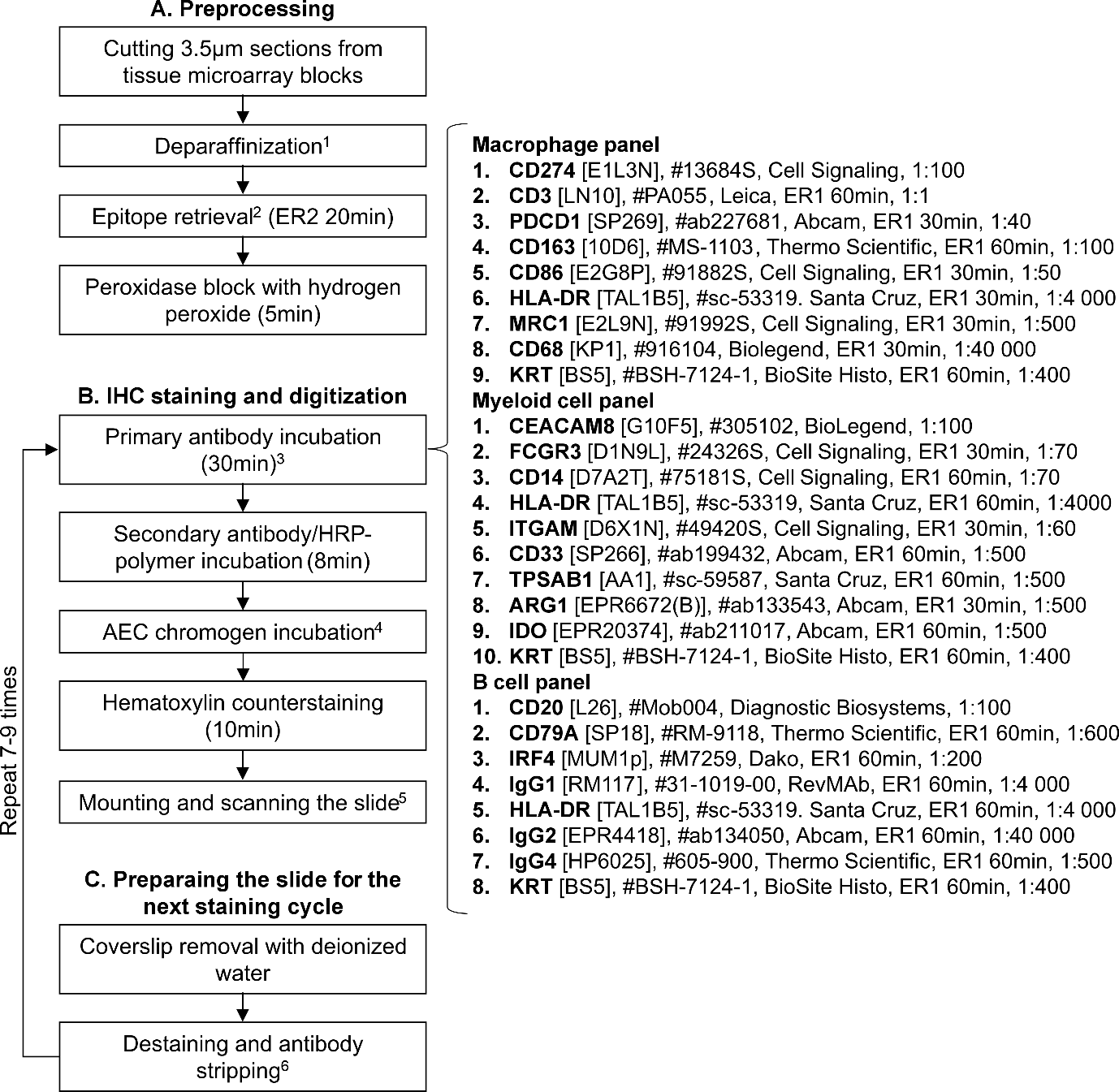


**Figure S3. Multiplex immunohistochemistry protocol.** Tissue microarray sections were stained 8 times (B cell panel), 9 times (Macrophage panel), or 10 times (myeloid cell panel) sequentially using an automated immunohistochemistry stainer. Incubations were conducted at room temperature if not otherwise specified. Abbreviations: IHC, immunohistochemistry; AEC, 3-Amino-9-ethylcarbazole

^1^ Dewax solution (AR9222, Leica Biosystems), 30 min, 60–72 ˚C.

^2^ Epitope retrieval done with BOND epitope retrieval solution 2 (EDTA based, pH9, AR9640, Leica Biosystems) with 20 min heating time.

^3^ Primary antibodies (name, clone, catalogue number and manufacturer) together with the selected antigen retrieval conditions and used dilutions are listed in the staining order.

^4^ AEC + high sensitivity substrate (K3469, Dako).

^5^ VectaMount AQ Aqueous Mounting Medium (H-5501, Vector Laboratories), digitized with a 20x objective using NanoZoomer XR (Hamamatsu) slide scanner.

^6^ The sections were destained with ethanol and antibody stripping was done with heat-induced epitope retrieval using BOND epitope retrieval solution 1 (citrate based, pH 6, AR9961, Leica Biosystems) or 2. Suitable epitope retrieval conditions were optimized for each antibody.


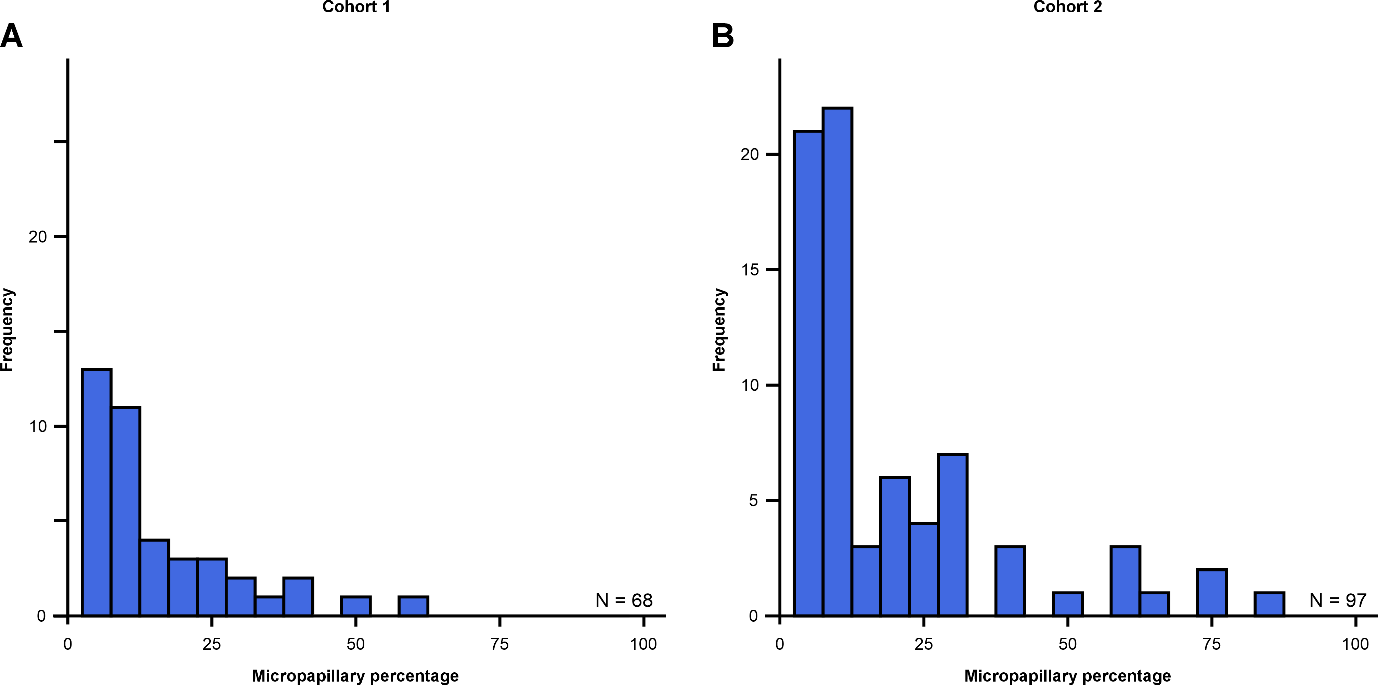


**Figure S4. Distributions of micropapillary growth pattern percentages.** Frequencies of micropapillary growth pattern in colorectal cancers with a micropapillary component in (A) Cohort 1 and (B) Cohort 2.


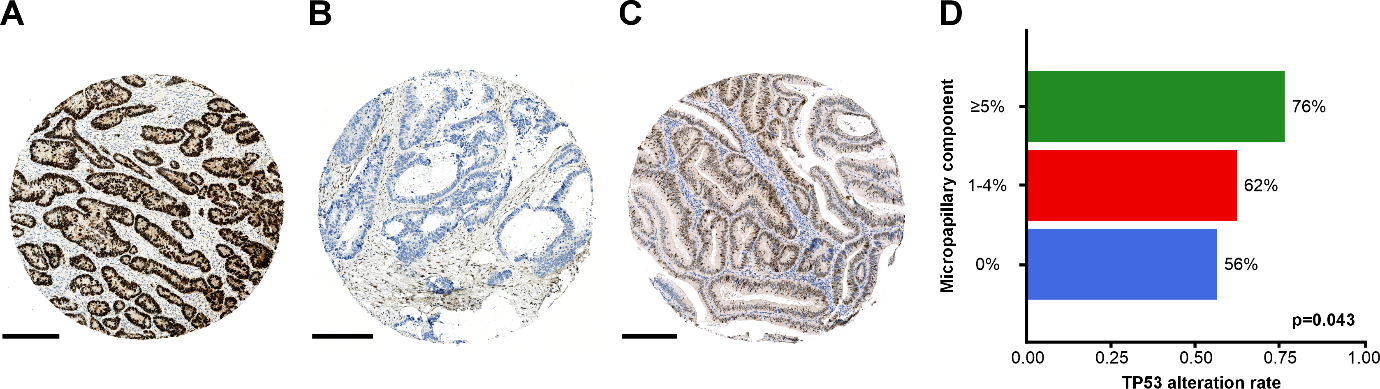


**Figure S5. TP53 alterations in micropapillary colorectal adenocarcinoma.** (A,B) Example tissue microarray cores of mutation-type TP53 expression patterns (A: diffuse overexpression; B: absent). (C) Example tissue microarray core of wildtype TP53 expression pattern (heterogenous). (D) Bar chart of the frequency of TP53 alteration according to micropapillary growth pattern in colorectal cancer. The analysis is based on Cohort 1 (*N* = 760). Scale bar, 250 µm.


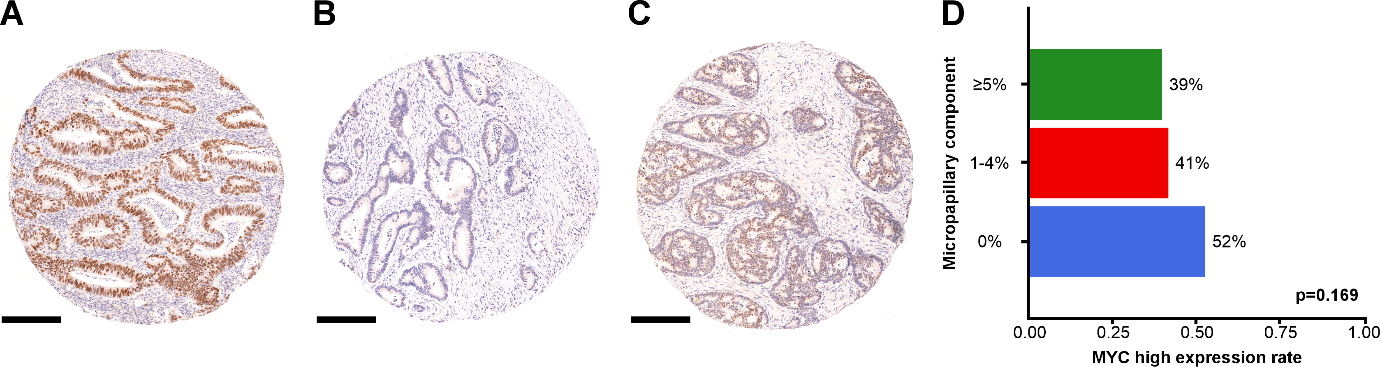


**Figure S6. MYC expression in micropapillary colorectal adenocarcinoma.** (A) Example tissue microarray core of very high MYC expression. Nuclear immunolocalization can be seen in nearly all cancer cells. (B) Example tissue microarray core of very low MYC expression. Less than 10% of cancer cells show nuclear staining. (C) Example tissue microarray core of colorectal cancer with average MYC expression. Nuclear staining is visible in ~50% of cancer cells. (D) Bar chart of the frequency of MYC high (>50%) colorectal cancers according to micropapillary growth pattern. Panel (D) is based on Cohort 1 (*N* = 760). Scale bar, 250 µm.


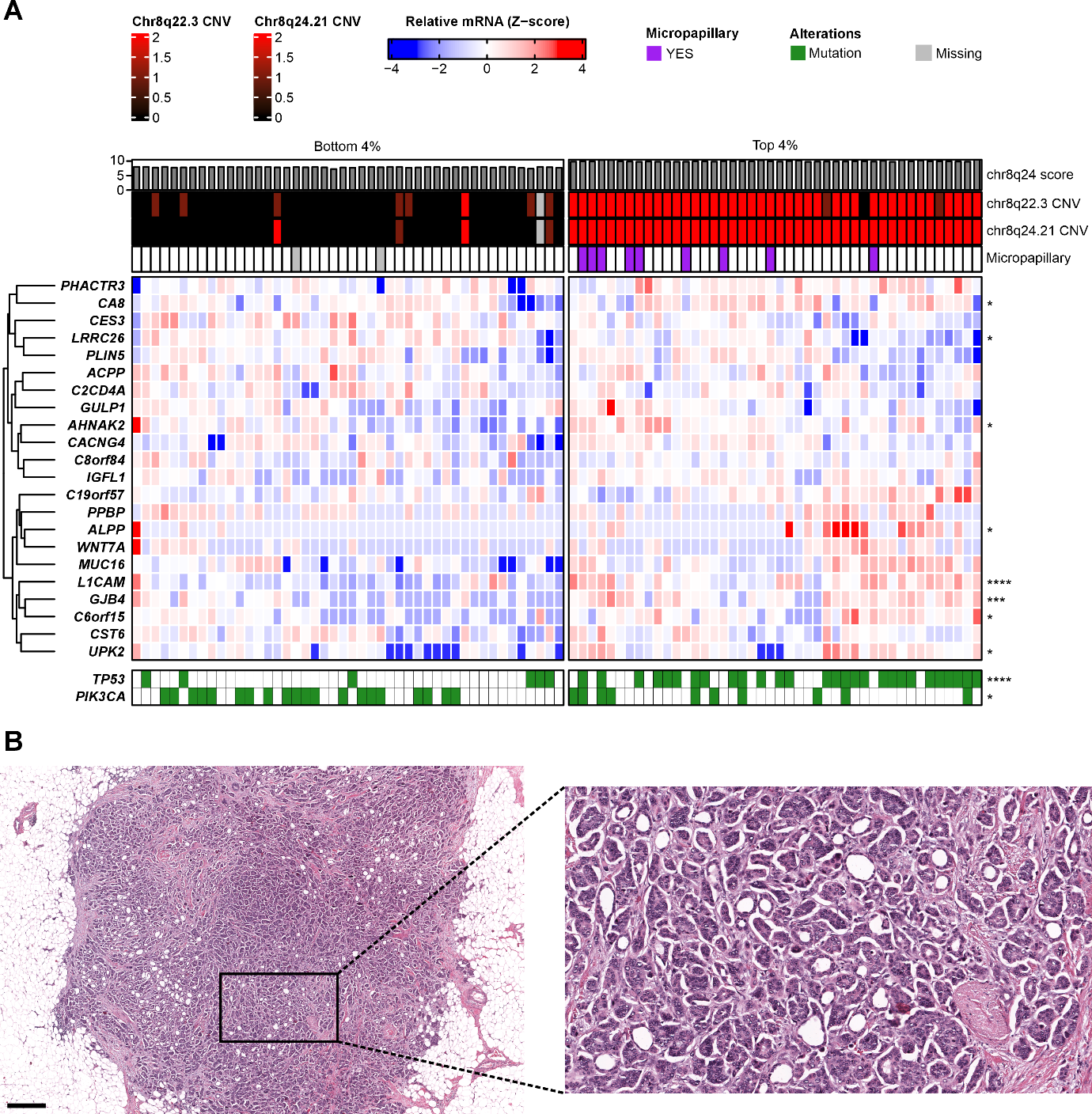


**Figure S7. Chromosome region 8q24 gene expression score analysis of TCGA BRCA cohort cases.** (A) Heatmap showing features of cases among top and bottom 4% of Chr8q24 gene-expression score. (B) Hematoxylin & eosin (H&E)-stained section of invasive micropapillary carcinoma of the breast (TCGA-BH-A0H6). In the close-up magnification, micropapillary structures can be seen with the customary retraction artifacts, resembling the morphologic findings of micropapillary colorectal adenocarcinomas. **p*-value<0.05, ***p*-value<0.01, ****p*-value<0.001, *****p*-value<0.0001. Scale bar, 400 µm. Abbreviations: CNV, copy number variation

**Supplementary Tables S1–S3**

**Table S1.** Genes used for the Chr8q24 locus gene expression score in the breast cancer cohort analysis

| *FAM83A* | *FBXO32* | *TOP1MT* |
| --- | --- | --- |
| *PSCA* | *MAL2* | *FOXH1* |
| *KCNK9* | *SAMD12* | *POU5F1B* |
| *TNFRSF11B* | *TMEM65* | *ZNF251* |
| *COL22A1* | *SQLE* | *SLC39A4* |
| *LY6E* | *LY6D* | *NSMCE2* |
| *KLHL38* | *TRIB1* | *ZHX2* |
| *PLEC* | *TRMT12* | *EFR3A* |
| *ZNF34* | *CHRAC1* | *SLC45A4* |
| *TATDN1* | *ZFP41* | *ZNF623* |
| *C8orf33* | *EEF1D* | *TSNARE1* |
| *TRAPPC9* | *RAD21* | *RPL8* |
| *FAM83H* | *ZNF707* | *SNTB1* |
| *ST3GAL1* | *NRBP2* | *EXT1* |
| *PTK2* | *MTBP* | *DERL1* |
| *EIF3H* | *PVT1* | *KHDRBS3* |
| *TAF2* | *HSF1* | *ZNF696* |
| *FAM91A1* | *GRINA* | *ZNF7* |
| *RNF139* | *PHF20L1* | *ZNF250* |
| *MED30* | *ARC* | *DSCC1* |
| *UTP23* | *DENND3* |  |

**Table S2.** Multivariable Cox proportional hazards regression models for cancer-specific survival and overall survival in Cohorts 1 and 2

|  | Cohort 1 | | |  | Cohort 2 | |  |
| --- | --- | --- | --- | --- | --- | --- | --- |
|  | Cancer-specific survival | Overall  survival | Cancer-specific survival | | | Overall  survival | |
|  | Multivariable  HR (95% CI) | Multivariable  HR (95% CI) | Multivariable  HR (95% CI) | | | Multivariable  HR (95% CI) | |
| Micropapillary growth pattern |  |  |  | | |  | |
| 0% | 1 (referent) | 1 (referent) | 1 (referent) | | | 1 (referent) | |
| 1–4% | 1.88 (0.85–4.13) | 1.36 (0.69–2.68) | 1.11 (0.58–2.12) | | | 0.96 (0.53–1.72) | |
| ≥5% | 2.57 (1.52–4.32) | 1.76 (1.08–2.87) | 1.24 (0.84–1.82) | | | 1.47 (1.08–2.00) | |
| Age |  |  |  | | |  | |
| <65 | 1 (referent) | 1 (referent) | 1 (referent) | | | 1 (referent) | |
| 65–75 | 1.46 (0.93–2.29) | 1.56 (1.07–2.27) | 1.15 (0.85–1.55) | | | 1.31 (1.02–1.68) | |
| >75 | 2.55 (1.63–3.98) | 4.28 (3.02–6.08) | 1.82 (1.36–2.43) | | | 2.93 (2.33–3.69) | |
| Sex |  |  |  | | |  | |
| Male | 1 (referent) | 1 (referent) | 1 (referent) | | | 1 (referent) | |
| Female | 0.92 (0.65–1.31) | 0.81 (0.62–1.04) | 0.86 (0.68–1.09) | | | 0.75 (0.62–0.89) | |
| Year of operation |  |  |  | | |  | |
| 2000–2005 | – | – | 1 (referent) | | | 1 (referent) | |
| 2006–2010 | 1 (referent) | 1 (referent) | 0.63 (0.48–0.84) | | | 0.72 (0.58–0.88) | |
| 2011–2015 | 0.95 (0.63–1.43) | 0.88 (0.65–1.19) | 0.51 (0.38–0.68) | | | 0.64 (0.52–0.80) | |
| 2016–2020 | 0.42 (0.27–0.68) | 0.53 (0.37–0.74) | – | | | – | |
| Tumor location |  |  |  | | |  | |
| Proximal colon | 1 (referent) | 1 (referent) | 1 (referent) | | | 1 (referent) | |
| Distal colon | 1.22 (0.78–1.92) | 1.13 (0.80–1.60) | 0.88 (0.68–1.15) | | | 0.93 (0.76–1.14) | |
| Rectum | 0.96 (0.61–1.49) | 1.09 (0.78–1.53) | 0.81 (0.56–1.16) | | | 0.86 (0.65–1.13) | |
| UICC disease stage |  |  |  | | |  | |
| I–II | 1 (referent) | 1 (referent) | 1 (referent) | | | 1 (referent) | |
| III | 3.03 (1.74–5.28) | 1.27 (0.91–1.78) | 3.25 (2.37–4.46) | | | 1.57 (1.28–1.93) | |
| IV | 23.16 (12.95–41.41) | 8.18 (5.58–12.00) | 18.75 (13.40–26.22) | | | 8.04 (6.30–10.26) | |
| Lymphovascular invasion |  |  |  | | |  | |
| No | 1 (referent) | 1 (referent) | 1 (referent) | | | 1 (referent) | |
| Yes | 1.77 (1.12–2.80) | 1.39 (1.03–1.89) | 1.94 (1.50–2.51) | | | 1.60 (1.30–1.96) | |
| MMR status |  |  |  | | |  | |
| MMR proficient | 1 (referent) | 1 (referent) | 1 (referent) | | | 1 (referent) | |
| MMR deficient | 0.49 (0.23–1.06) | 0.93 (0.59–1.47) | 0.67 (0.40–1.10) | | | 0.82 (0.58–1.15) | |
| *BRAF* mutation |  |  |  | | |  | |
| Wild-type | 1 (referent) | 1 (referent) | 1 (referent) | | | 1 (referent) | |
| Mutant | 2.01 (1.12–3.62) | 1.52 (0.99–2.34) | 1.41 (0.92–2.16) | | | 1.55 (1.13–2.12) | |
| *HER2* amplification |  |  |  | | |  | |
| No | 1 (referent) | 1 (referent) | 1 (referent) | | | 1 (referent) | |
| Yes | 0.67 (0.21–2.12) | 1.20 (0.63–2.29) | 0.71 (0.35–1.44) | | | 0.88 (0.51–1.53) | |

Abbreviations: CI, confidence interval; HR, hazard ratio; UICC, Union for International Cancer Control; MMR, mismatch repair.

**Table S3.** Associations of lymphocytic reaction patterns with the micropapillary growth pattern in Cohort 2 (*N* = 1,100)

|  |  | Micropapillary growth pattern | | |  |
| --- | --- | --- | --- | --- | --- |
| Characteristic | Total | 0% | 1–4% | ≥5% | *p* value |
| Peritumoral reaction |  |  |  |  | **0.0005** |
| Absent | 173 (16%) | 146 (84%) | 5 (2.9%) | 22 (13%) |  |
| Low | 427 (39%) | 381 (89%) | 15 (3.5%) | 31 (7.3%) |  |
| Intermediate | 411 (37%) | 389 (89%) | 7 (1.7%) | 15 (3.6%) |  |
| High | 89 (8.1%) | 87 (98%) | 0 (0.0%) | 2 (2.2%) |  |
| Intratumoral periglandular reaction |  |  |  |  | **<0.0001** |
| Absent | 168 (15%) | 133 (79%) | 7 (4.2%) | 28 (17%) |  |
| Low | 414 (38%) | 375 (91%) | 12 (2.9%) | 27 (6.5%) |  |
| Intermediate | 454 (41%) | 432 (95%) | 8 (1.8%) | 14 (3.1%) |  |
| High | 64 (5.8%) | 63 (98%) | 0 (0.0%) | 1 (1.6%) |  |
| Tumor-infiltrating lymphocytes |  |  |  |  | **<0.0001** |
| Absent | 640 (58%) | 561 (88%) | 20 (3.1%) | 59 (9.2%) |  |
| Low | 304 (28%) | 289 (95%) | 7 (2.3%) | 8 (2.6%) |  |
| Intermediate | 118 (11%) | 115 (97%) | 0 (0.0%) | 3 (2.5%) |  |
| High | 38 (3.5%) | 38 (100%) | 0 (0.0%) | 0 (0.0%) |  |
| Crohn’s-like lymphoid reaction |  |  |  |  | **0.007** |
| Absent | 334 (30%) | 296 (89%) | 10 (3.0%) | 28 (8.4%) |  |
| Low | 444 (41%) | 396 (89%) | 14 (3.2%) | 34 (7.7%) |  |
| Intermediate | 223 (20%) | 216 (97%) | 3 (1.3%) | 4 (1.8%) |  |
| High | 99 (9.0%) | 95 (96%) | 0 (0.0%) | 4 (4.0%) |  |
|  |  |  |  |  |  |

*P*-values in bold are statistically significant.
